# A CAR/CD19 Jurkat cell model for bioprocess screening and expansion: potential use for CAR-T cells production

**DOI:** 10.3389/fbioe.2026.1917849

**Published:** 2026-10-07

**Authors:** Hugo E. Fuentes-Bustos, Alejandrina Hernández-López, Marta Zapata-Tarrés, Juan Carlos Bustamante-Ogando, Blanca Angélica Segura-Pacheco, Alberto Olaya-Vargas, Angélica Meneses-Acosta

**Affiliations:** 1 Pharmaceutical Biotechnology Laboratory, Faculty of Pharmacy, Universidad Autónoma del Estado de Morelos, Cuernavaca, Morelos, Mexico; 2 Secretaría de Ciencia, Humanidades, Tecnología e Innovación, SECIHTI, Mexico City, Mexico; 3 Comisión Coordinadora de Institutos Nacionales de Salud y Hospitales de Alta Especialidad (CCINSHAE), Ministry of Health (Secretaría de Salud), Mexico City, Mexico; 4 Immunodeficiencies Research Laboratory and Clinical Immunology Department, Instituto Nacional de Pediatría, Mexico City, Mexico; 5 Department of Research and Development, Triovance SAPI de CV, Mexico City, Mexico; 6 Hematopoietic Stem Cell Transplantation and Cell Therapy Program, Instituto Nacional de Pediatría, Mexico City, Mexico

**Keywords:** Jurkat cell model, expansion optimization, cell expansion, culture media, modes of culture operation, cell adoptive therapies

## Abstract

**Introduction:**

Cell expansion is one of the most critical steps in the development of CAR-T cell manufacturing processes because it directly influences cell yield and process performance. The systematic optimization of expansion parameters using primary human T cells is often limited by inter-donor variability, cost, and experimental complexity. So, it is necessary to study more homogeneous populations that allow to stablish standardized conditions in a controlled environment that were able to be used as standard. So, in this study, we developed a Jurkat CAR-CD19 cell-based platform as a reproducible model for optimizing the cell expansion bioprocess under controlled culture conditions.

**Methods:**

CAR-CD19-expressing Jurkat cells were pre-generated, molecularly characterized and subsequently used to evaluate key bioprocess variables, including glucose concentration, inoculum density, culture medium composition (RPMI + 10% FBS versus Nutri-T without serum), shaking speed, and different culture operating modes (batch, fed-batch, and semi-perfusion). Cell growth kinetics, metabolite consumption, metabolite production, and CAR expression were evaluated throughout the optimization process.

**Results:**

An intermediate glucose concentration supported cell expansion without additional benefit from higher glucose supplementation. Serum-free Nutri-T medium promoted higher viable cell densities than RPMI (used as control media) under the evaluated conditions. Among the operating modes evaluated, semi-perfusion achieved the highest viable cell density, maintaining high cell viability and a homogeneous CAR-positive cell population at the end of culture in Nutri-T media.

**Conclusion:**

These findings demonstrate that the Jurkat CAR-CD19 model provides a robust and reproducible experimental platform for optimizing cell expansion bioprocesses. While further validation in primary human CAR-T cells is required, this model allows for the systematic evaluation of culture variables, minimizing experimental variability and facilitating early-stage process development for adoptive cell therapies.

## Introduction

CAR-T cell therapy has established itself as a treatment modality for various hematologic malignancies and is being actively investigated for the treatment of solid tumors and other diseases (Zhou et al., 2024). Despite these notable clinical advances, its widespread implementation remains limited by the complexity, cost (ranging from $373,000 to $475,000), and variability of current manufacturing workflows (Hernández-López et al., 2021). Consequently, considerable effort has been devoted to developing more efficient manufacturing strategies capable of improving process robustness, reducing production costs, and increasing patient access to this therapy. However, cell expansion represents one of the most critical stages of the process, as it accounts for the majority of the production time while generating the dose of therapeutic cells required for infusion. Depending on the commercial product, this phase typically requires approximately 12–14 days to yield between 1 and 6 × 10^8^ CAR-positive T cells (Zhou et al., 2024; Petersen et al., 2018; Costariol et al., 2020). Beyond achieving a sufficient number of cells, expansion conditions directly influence over the critical product quality attributes (such as cell phenotype, metabolic fitness, proliferative capacity, and differentiation state) making this stage a decisive factor in the overall process performance. Consequently, optimizing cell expansion in biorelevant model systems poses a challenge, given that autologous T cells are often obtained from patients who have undergone intensive prior treatments and whose proliferative capacity is compromised (Castella et al., 2020). These biological limitations are accompanied by higher manufacturing costs and a longer total process time (from extraction to infusion) (Costariol et al., 2020; Li et al., 2024). Prolonged expansion periods can promote progressive T-cell differentiation and premature exhaustion, ultimately reducing therapeutic efficacy and post-infusion persistence highlighting the need for efficient bioprocesses that maximize cell expansion while preserving cell quality (Amini et al., 2020; Song et al., 2024; Amini et al., 2020; Song et al., 2024).

Given that cell expansion constitutes one of the most influential unit operations within the CAR-T cell manufacturing workflow, optimizing this stage requires more than simply maximizing cell proliferation due to the mentioned effects on the critical quality attributes of the final cell product (Ayala et al., 2024; Reddy et al., 2020). Recent advances in CAR-T cell manufacturing have placed increasing emphasis on the systematic development of bioprocesses, whereby key process variables are rationally optimized to improve manufacturing reliability, reproducibility, and product quality (Abou-El-Enein et al., 2021; Matos et al., 2026). Rather than evaluating individual culture parameters in isolation, this approach promotes the integrated assessment of variables such as culture medium composition, nutrient availability, inoculum density, agitation conditions, and operational strategies, with the aim of establishing a consistent framework and reproducible cell expansion processes that support rational bioprocess development (García-Aponte et al., 2021; Rostamian et al., 2022; Ma et al., 2024; Sudarsanam et al., 2022). To this day, there is a growing field focused on exploring the most suitable culture parameters following the current necessities regarding manufacturing challenges and regulation concerning the biosafety of the final products. For instance, according to the European Medicines Agency (EMA) the use of Fetal Bovine Serum (FBS) in the manufacture of human biological medicinal products is a major concern related to biosafety due to the extraneous agents, the batch-to-batch variability and the risks regarding adventitious agents, resulting in the need of media formulations that comply the safety regulations (European Medicines Agency, 2013). Meanwhile, the culture parameters have been variables that historically can be exploited in the manufacture of biologicals (Sudarsanam et al., 2022). Nonetheless, additional considerations must be taken while working with primary T cells. As it was mentioned above, implementing this systematic optimization strategy directly in autologous T cells remains experimentally challenging due to the inherent biological variability associated with patient-derived cells, heterogeneous activation states, limited T cell availability, high manufacturing costs and ethics considerations restrict the evaluation of multiple culture variables under controlled and reproducible conditions (Colao et al., 2026; Fesnak, 2020). As a result, it is inherently difficult to distinguish the individual contribution of specific culture parameters in the expansion performance, highlighting the need for suitable experimental platforms that enable controlled process development during the early stages. In this context, the use of biological relevant *in vitro* models has been recognized as a suitable strategy during the non-clinical development of advanced therapy medicinal products. The recent European Medicines Agency (EMA) guideline on quality, non-clinical, and clinical requirements for investigational advanced therapy medicinal products recommends conducting non-clinical studies using the most pharmacologically relevant *in vitro* and/or *in vivo* models available, in order to establish proof-of-concept and support the assessment of efficacy and safety prior to clinical translation (European Medicines Agency, 2025). Consequently, reproducible cell models (capable of minimizing biological variability while preserving key biological characteristics) constitute valuable tools for the systematic development and optimization of bioprocesses.

Thus, among the available T-cell models, the Jurkat E6.1 cell line, an immortalized T-cell line originally isolated from a 14-year-old donor with acute lymphoblastic leukemia (ALL) has been widely used to investigate T-cell activation, receptor signaling, genetic engineering strategies, and CAR functionality, owing to its homogeneous genetic background, reproducible proliferative behavior, suspension growth, and high susceptibility to genetic modification (Colao et al., 2026; Bartelt et al., 2009; Subham et al., 2023). These characteristics facilitate the systematic evaluation of culture conditions in standardized experimental settings while minimizing biological variability (Colao et al., 2026). Furthermore, various studies have successfully generated Jurkat models expressing CARs targeting antigens such as CD19, CD22, HER3, EGFR, and prostate cancer-associated antigens, demonstrating the platform’s versatility for evaluating CAR design, antigen recognition, signaling, and functional responses. Beyond mechanistic studies, Jurkat-based CAR models have also been proposed as rapid, cost-effective, and high-throughput experimental platforms for an early-stage optimization prior to validation in primary human T cells (Lainšček et al., 2023; Aoyama et al., 2022; Hassani et al., 2020; Bloemberg et al., 2020; Pipiya et al., 2024). Therefore, rather than replacing primary CAR-T cells, CAR-expressing Jurkat cells offer a reproducible surrogate platform for early stage systematic bioprocess development, enabling controlled evaluation of culture variables before translation to biologically more complex cellular systems.

Although previous studies have successfully employed Jurkat-based CAR models for receptor engineering and functional characterization (Lainšček et al., 2023; Aoyama et al., 2022; Hassani et al., 2020; Bloemberg et al., 2020; Pipiya et al., 2024), their application as reproducible platforms for the systematic integration of culture medium selection, nutrient optimization, inoculum density, operational strategies, and metabolic characterization during bioprocess development remains limited. Consequently, this study aimed to establish a platform based on CAR-CD19 Jurkat cells model for the systematic development of bioprocesses, integrating the sequential optimization of culture medium composition, glucose concentration, inoculum density, agitation conditions, operational culture strategies, and metabolic characterization.

Thus, to identify culture conditions associated with improved cell expansion performance under reproducible experimental conditions while mainly assessing the quality parameters related to the bioprocess and identity of the CAR-CD19 expressing Jurkat cell line.

## Materials and methods

### Cell lines and cell culture

Jurkat cells Clone E6-1 (ATCC® TIB-152™) were obtained from the American Type Culture Collection (ATCC; Manassas, VA, USA). The chimeric antigen receptor (CAR) construct comprised a murine-derived single-chain variable fragment (scFv) specific for human CD19 (FMC63), linked to CD28 hinge and transmembrane domain, followed by the intracellular costimulatory domain of 4-1BB (CD137) and the CD3ζ signaling domain, under constitutive control of the EF1α promoter. The construct was delivered via lentiviral transduction at a multiplicity of infection (MOI) of 8. Transduced cells were selected by antibiotic resistance (Puromycin) and sorted by flow cytometry for scFv, yielding a polyclonal Jurkat/CAR-CD19 population at passage 8.

Jurkat cells Clone E6-1 (parental Jurkat cells) and transduced Jurkat cells (Jurkat/CAR-CD19) were grown in RPMI-1640 as standard medium with FBS [Sigma-Aldrich, catalog No. R6504, and supplemented with 10 mM HEPES, 4.5 g/L D-glucose, 1 mM sodium pyruvate, 2 mM L-glutamine, and 1.5 g/L sodium bicarbonate, 10% (v/v) heat-inactivated fetal bovine serum (FBS, Gibco)]. In addition, both cell lines were adapted to grow in 4Cell® Nutri-T medium (defined GMP medium without FBS) (Sartorius, Beit Haemek, Israel) over nine passages before initiating expansion experiments. All cultures were maintained in a humidified incubator (New Brunswick™ S41i, Eppendorf AG, Germany) at 37 °C with 10% CO_2_ without agitation.

### Growth kinetics setup

Pre-expansion to generate the inoculum was performed in T75 static flasks (Item No. 430641U, Corning Inc.) with a nominal volume of 250 mL and a working volume of 10–30 mL. This pre-expansion procedure was made in every growth kinetic.

All growth kinetics experiments on different modes of operation were carried out in 125 mL Erlenmeyer flasks (Cat. No. 431143, Corning Inc.) by biological duplicates with a working volume of 25 mL at a specific initial seeding density. Cultures were maintained at 37 °C in a humidified incubator with 10% CO_2_ and orbital shaking at 40 and 130 rpm using a platform with 2.5 cm orbital eccentricity.

### Batch growth kinetics

Starting with the parental linage (Jurkat cell line), three levels of glucose were evaluated on static mode, starting with 2 g/L (low), 4.5 g/L (medium/standard) and 6.5 g/L (high) as well as two different inoculum (1 and 2 × 10^5^ VC/mL) to establish the best cell culture setup for the growth kinetics. Once determined, a factorial design was used to construct an experimental matrix for stirring batch-mode kinetics. The matrix included two factors: The first factor was the type of medium cell culture with two levels: RPMI + 10% FBS and free serum media Nutri-T; the second factor was the agitation level at two levels [40 rpm (medium agitation) and 130 rpm (high agitation)]. Four combinations were evaluated using the parental cell line to characterize growth and metabolic profiles. Based on the expansion related calculations mentioned above, a subsequent culture was conducted using 130 rpm in Nutri-T media in the Jurkat/CAR-CD19 modified lineage as the condition with the highest growth. Finally, cell proliferation, metabolic consumption, and specific growth/production rates were determined and used to elaborate feeding strategies for fed-batch and semi-perfusion experiments.

### Fed-batch growth kinetics

Based on the obtained metabolic data from the growth kinetic cultures by batch, the three fed-batch strategies were made differing by the starting feeding day, being at 96 h (early exponential phase) for the first fed-batch strategy (FB1), at 144 h (late exponential phase) for the second strategy (FB2) and at 120 h (mid-exponential phase) for the third strategy (FB3).

In the feeding strategy, the used feeding media was the basal Nutri-T. After an initial batch phase of 72, 120, and 96 h respectively with an initial volume of 14 mL for each strategy, the bolus feed volume equivalent to 14% (3.6 mL, according to the calculation of nutrient consumption) of the final working volume (25 mL) was added manually on a three-day feeding schedule. Although continuous feeding strategies have demonstrated positive outcomes and are known to extend culture duration more effectively than bolus feeding, the bolus approach was selected in this study to establish and monitor an efficient and rapid expansion strategy. The feeding volume was calculated based on a simple system of adding/diluting moles of the four metabolites analyzed in the cell culture media of the batch cultures (static/Nutri-T and 130 rpm/Nutri-T) at different points of the exponential phase. The target concentrations of glucose, glutamine, lactate and glutamate with the established feeding volume were estimated based on the expected addition/dilution of the metabolites: 28–55 mmol/L, >2 mmol/L, <40 mmol/L and <1 mmol/L respectively.

Later, daily samples were analyzed to assess the metabolic profiles in each fed-batch strategy.

### Semi-perfusion growth kinetics

Based on the results of metabolite consumption during batch kinetics as well as the observed behaviors in the different feeding points at the fed-batch mode, the semi-perfusion strategy was made. The daily media exchange started at the mid-exponential phase, with an exchange of 50% of the total volume which allowed the depletion of metabolic waste by half. Therefore, it consisted of an exchange of 50% of the total culture medium (12.5 mL) in the mid-exponential phase, managing a continuous period (daily) by using the basal Nutri-T medium. The strategy was calculated as it was previously described for fed-batch cultures. However, given the increased metabolic demand because of constant cell growth, the semi-perfusion strategy was adapted on the methodology described by Mizukami and Swiech (2020), which made daily media exchanges proportionally to the cell viable concentration. Specifically, no medium exchange was performed when cell density was < 2 × 10^6^ VC/mL; 50% of the culture volume (12.5 mL) was exchanged at 2–10 × 10^6^ VC/mL; 75% (18.75 mL) at 10–15 × 10^6^ VC/mL; and 100% (25 mL) at densities >15 × 10^6^ VC/mL according to (Mizukami and Swiech, 2020).

Medium changes were made manually from the culture flasks by removing the respective percentage of culture medium based on the total volume (25 mL), which then was centrifuged at 1,400 rpm for 5 min. Cell recovery following individual centrifugation and medium-exchange operations was not independently quantified. The supernatant was then removed, and the cellular pellet were resuspended in Nutri-T basal medium with the same volume that was removed before finally returning them to the culture flasks. Between this process, daily samples were taken to analyze the cell viability and their metabolic profile. Because this approach does not involve continuous medium flow or a dedicated cell-retention device, it is referred to throughout the manuscript as semi-perfusion operation mode.

### Expression stability assays

CAR expression stability was determined by flow cytometry analysis using an F(ab')2 Anti-Human IgG-(Fab')2 (PE) antibody (Abcam Cat. No. ab98606). Flow cytometry staining procedure was developed on Jurkat E6-1 and Jurkat CAR/CD19 cells. The number of cells was adjusted to 1 × 10^6^ cells/mL for staining. A cell suspension (100 μL) was incubated with Goat F(ab')2 Anti-Human IgG - (Fab')2 (PE) antibody (Abcam Cat. No. ab98606) according to the manufacturer’s instructions and washed twice with PBS and then re-suspended in PBS and analyzed by flow cytometry. PE-fluorescent cells were regarded as CAR-positive. Because cells were fixed immediately after sample collection and no viability dye was used, live/dead discrimination was not performed. The gating strategy therefore consisted of singlet discrimination, FSC/SSC-based cellular population selection, and PE-based identification of CAR-positive cells (Supplementary Figure S1).

Unstained parental Jurkat cells, stained parental Jurkat cells, unstained Jurkat/CAR-CD19 cells, and stained Jurkat/CAR-CD19 cells were included as fluorescence controls. Unstained Jurkat/CAR-CD19 cells were used to define the baseline fluorescence distribution of the CAR-expressing cell line (SF1).

Samples were collected at the selected late-exponential phase and immediately fixed prior to flow-cytometric analysis. Approximately 10,000 events were acquired for each sample. Singlet discrimination was performed using FSC-A versus FSC-H, followed by selection of the cellular population using FSC-A versus SSC-A. CAR-positive cells were identified according to PE fluorescence relative to the corresponding unstained controls. The percentage of CAR-positive cells was calculated relative to the cellular population subjected to CAR-expression analysis. Median fluorescence intensity (MFI) was calculated as the median PE-A fluorescence of events within the CAR-positive population (ST3).

Data acquisition was performed on BD FACS Canto II (BD Biosciences) flow cytometer and data was analyzed with Winlist 3D v 9.0.1 software (Verity software House).

### Analytical techniques

A 1 mL sample was collected daily for cell counting and culture monitoring. The removed volume was immediately replaced with an equivalent volume of fresh medium; therefore, daily sampling did not result in a progressive reduction of the culture volume. The cell counting was made by employing the trypan blue exclusion technique in a hemacytometer chamber (Neubauer chamber). The calculated cellular expansion parameters were cellular viability (%), specific growth rate (μ), cell viable density (VC/mL), total cell density, and fold expansion. Total viable cell number was calculated from viable cell concentration and the corresponding cell culture volume. Fold expansion was calculated as the ratio between the final total viable cell number and the initial total viable cell number. Maximum total viable cell yield was determined at the point of maximum viable cell concentration for each culture condition. Cumulative population doublings were calculated from the initial and final total viable cell numbers. Doubling time was calculated independently from the exponential phase of the growth kinetics using at least three data points.

All measurements were made in technical triplicates. Then, the samples were used to analyze the daily metabolic concentrations of glucose (Glu), glutamine (Gln), lactate (Lac), and Glutamate (Glu) in a biochemical analyzer (YSI-2950 Life Sciences). Once the growth and metabolic data was calculated, the specific consumption/production rate was determined at pmol/cell-day.

Viable cell density:

$$\frac{VC}{mL}=\left(\frac{viable\;cell\;number}{number\;of\;cuadrants}\;*\;1x10^{4}\right)\;DF$$

Cellular viability (%):

$$V\text{iability }\left(\%\right)=\frac{Number\;of\;viable\;cells}{Viable\;cells+non-viable\;cells}\;*\;100$$

Fold expansion:

$$\text{Fold expansion}=\frac{Maximun\;viable\;cell\;number}{Initial\;viable\;cell\;number}$$

Cumulative population doublings

$$PD={\mathrm{Log}}_{2}\frac{Number\;of\;total\;final\;cells}{Number\;of\;initial\;total\;cells}$$

Specific growth rate:

$$\mu =\frac{\left(\ln\left(\frac{X\;\left(t\right)}{X\;\left(0\right)}\right)\right)}{\Delta t}$$

Doubling time:

$$td=\frac{\ln2}{\mu }$$

Specific consumption/production rate:

$$q_{s/p}=\frac{\Delta_{s/p}}{X_{avg}.\Delta t}$$

Percentage of positive Jurkat/CAR-CD19 cells:

$$\%CAR/CD19+=\frac{CAR\;positive\;events}{FSC-SSC\;cellular\;population\;events}X\;100$$

Where:

DF: Dilution factor.

X (t): Cell number at the end of the exponential phase.

X (o): Cell number at the start of the exponential phase.

Δt: Duration of the exponential phase.

Δ s/p = Final metabolite concentration–Initial metabolite concentration: Differential of metabolite concentration.

X_avg_: Average cell density.

### Quantitative data handling

All measurements were made by triplicate and represented as the mean values. GraphPad Prism 9 (GraphPad Software) was used to plot data and calculate statistics. P values meeting and = 0.05 level of significance are indicated in the figures. The precise statistical tests used are indicated in the figure legends. To evaluate the combinations between level of stirring and RPMI or Nutri-T medium on the parental cell line, an experimental matrix was created by using the Minitab version 17.1 program.

## Results

### Establishment of culture conditions for Jurkat/CAR-CD19 expansion

In this study, a chimeric antigen receptor (CAR) targeting CD19 was used. The CAR consists of an extracellular murine single-chain variable fragment (scFv) specific for CD19, linked to the hinge and transmembrane domains, the intracellular signaling modules by CD28, 4-1BB (CD137) and CD3ζ. Constitutive expression is driven by the elongation factor 1 alpha (EF1α) promoter to ensure high and stable surface CAR expression. This CAR-CD19 demonstrated antigen-specific recognition and potent cytotoxic activity against CD19-positive target cells (data not shown), with functional performance comparable to murine-based CAR constructs currently used in both clinical and preclinical settings.

### The effect of glucose supplementation on batch mode cultures

To establish culture conditions for subsequent expansion studies, Jurkat E6-1 cells were evaluated in two different culture media. Initially, RPMI-1640 supplemented with 10% FBS was used as the basal culture medium for Jurkat E6-1 cells. Subsequently, the cells were adapted to 4Cell Nutri-T medium (Sartorius) (a serum-free, GMP-compliant medium) over the course of nine passages. This adaptation was performed to establish serum-free culture conditions and reduce reliance on serum supplementation, while also providing a more defined culture environment for subsequent bioprocess optimization. Both culture conditions were then evaluated using Jurkat E6-1 and Jurkat/CAR-CD19 cell lines.

To determine the culture conditions for subsequent expansion studies, an experimental workflow was designed to sequentially evaluate glucose concentration, culture medium, and inoculum density, followed by further optimization of culture conditions (Figure 1A). Initially, parental Jurkat E6-1 cells were evaluated in RPMI-1640 medium supplemented with 10% fetal bovine serum (FBS) and three different glucose concentrations (Figure 1B). As observed in the figure, under low glucose concentration conditions (2 g/L), the cells exhibited the lowest expansion factor (27.67), whereas intermediate (4.5 g/L) and high glucose concentrations (6.5 g/L) favored greater expansion (42.95 and 43.45, respectively). No significant differences were observed between the intermediate and high concentration conditions, indicating that increasing glucose availability beyond 4.5 g/L did not further improve cell expansion under the conditions evaluated. Based on these results, growth kinetics were subsequently evaluated using glucose concentrations of 4.5 and 6.5 g/L (Figures 1C,D). With 4.5 g/L glucose, Jurkat E6-1 cells reached a maximum viable cell density of 4.3 ± 0.03 × 10^6^ VC/mL and a doubling time of 22.5 ± 0.75 h; viability remained stable during the exponential growth phase and declined gradually thereafter (Figure 1C). Increasing the glucose concentration to 6.5 g/L did not increase the maximum viable cell density (which remained comparable at 4.3 ± 0.34 × 10^6^ VC/mL) but resulted in a longer doubling time (28.7 ± 1.08 h) and a more pronounced decline in viability (Figure 1D). Consequently, RPMI medium with 4.5 g/L glucose was selected as the reference condition for subsequent experiments. To establish serum-free culture conditions, Jurkat E6-1 cells were subsequently adapted to 4Cell Nutri-T medium (Sartorius) over eight passages and evaluated under the same experimental framework (Figure 1E). Under this condition, the cells reached a maximum viable cell density of 5.98 ± 0.32 × 10^6^ VC/mL, with a doubling time of 23.1 ± 0.71 h. Although Nutri-T medium enabled a higher maximum cell density than RPMI medium under the conditions tested, viability dropped sharply after this peak density was reached. The use of this serum-free, established a more defined culture condition and reduced reliance on serum supplementation for subsequent bioprocess optimization. Based on these observations, the expansion of Jurkat/CAR-CD19 cells in RPMI medium containing 4.5 g/L glucose was compared with that in the serum-free medium Nutri-T, using an initial inoculum of 1.0 × 10^5^ viable cells/mL (Figure 1F). In RPMI, Jurkat/CAR-CD19 cells reached a maximum viable cell density of 4.63 ± 0.40 × 10^6^ VC/mL, with a doubling time of 26.65 ± 0.81 h. In contrast, Nutri-T medium enabled a higher maximum viable cell density of 6.87 ± 0.46 × 10^6^ VC/mL, although this was associated with a longer doubling time (38.29 ± 0.70 h) and a sharper decline in viability after reaching peak density. To further improve expansion under serum-free conditions, the initial inoculum was increased to 2.0 × 10^5^ VC/mL. Under these conditions, the cells reached a maximum viable cell density of 8.8 ± 0.68 × 10^6^ VC/mL and exhibited a shorter doubling time of 22.01 ± 0.39 h. However, viability subsequently dropped drastically, falling from >95% to <20% within 48 h. These results demonstrated that increasing the initial inoculum improved expansion kinetics and maximum cell density under serum-free conditions, while also highlighting the need for further optimization of culture performance.

**FIGURE 1:**
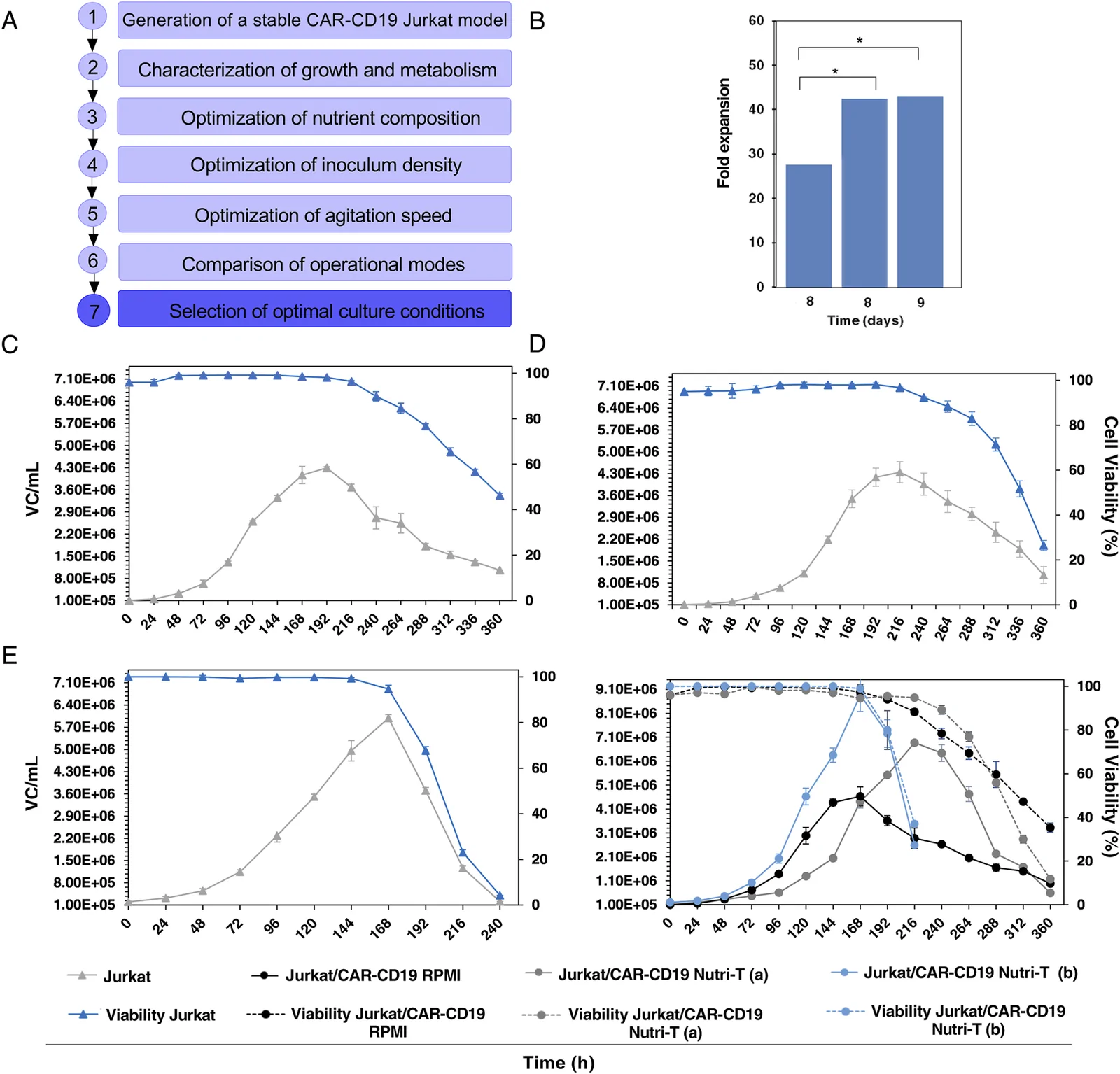
Establishment and characterization of the Jurkat/CAR-CD19 platform and initial evaluation of culture conditions. **(A)** General experimental workflow followed for the development and optimization of the Jurkat/CAR-CD19 cell expansion process, including the generation of a stable Jurkat/CAR-CD19 model, characterization of growth and metabolism, optimization of nutrient composition and inoculum density, evaluation of agitation and operational modes, and selection of culture conditions. **(B)** Fold expansion of Jurkat E6-1 cells cultured under low (2 g/L), intermediate (4.5 g/L), and high (6.5 g/L) glucose concentrations with 10% FBS. A markedly reduced expansion rate was observed under low glucose, whereas intermediate and high glucose supported higher proliferation with no significant difference between them (p < 0.05). **(C,D)** Growth kinetics of Jurkat E6-1 cells cultured under intermediate (4.5 g/L; panel **(C)** and high (6.5 g/L; panel **(D)** glucose concentrations. Both conditions reached maximum viable cell densities (4.3 × 106 VC/mL). **(E)** Growth kinetics of Jurkat E6-1 cells cultured in a serum-free, high-glucose medium (Nutri-T), achieving a higher maximum viable cell density of 5.9 × 106 VC/mL. **(F)** Growth kinetics of Jurkat/CAR-CD19 cells cultured under different media and inoculum conditions. Cells were grown in an intermediate-glucose medium (4.5 g/L) and in the serum-free, high-glucose Nutri-T medium with two initial inoculum densities: (a) 1.0 × 105 and (b) 2.0 × 105 VC/mL. The maximum viable cell density reached 4.6 × 106 VC/mL under intermediate glucose, and 6.8 × 106 and 8.8 × 106 cells/mL in Nutri-T medium for (a) and (b), respectively. (p < 0.05, unpaired Student’s t-test). Data are shown as mean ± SD from two biological replicates performed in technical triplicate.

### Effect of shaking speed and type of cell culture media in growth kinetics of Jurkat parental and Jurkat/CAR-CD19 cell lines

Figure 2 shows the growth kinetics of the parental cells carried out in two different culture media (RPMI and Nutri-T) under two shaking conditions (40 and 130 rpm). As observed, the different stages of growth are distinguished in each of the curves. The adaptation phase (lag) was approximately 72–96 h. The exponential phase lasts approximately 4 days (which varies between conditions in terms of the start time and its level of inclination), followed by the stationary phase, whose duration was around 48–72 h. Finally, the death phase is shown with an abrupt decrease between 20% and 30% in viability. Differences were identified in the duration of these phases between the evaluated media; in RPMI medium, cell viability remained above 95% up to 240 h, while in Nutri-T medium it remained around 75% during the same period. The better maximum cell densities achieved in each media were between 4–4.5 × 10^6^ VC/mL for RPMI medium, while Nutri-T media maintained a range between 5–6 x 10^6^ VC/mL regardless of the shaking speed in each case. This confirms that, under the evaluated conditions, Nutri-T medium promoted an additional expansion of between 1 and 2 × 10^6^ VC/mL compared to RPMI.

**FIGURE 2:**
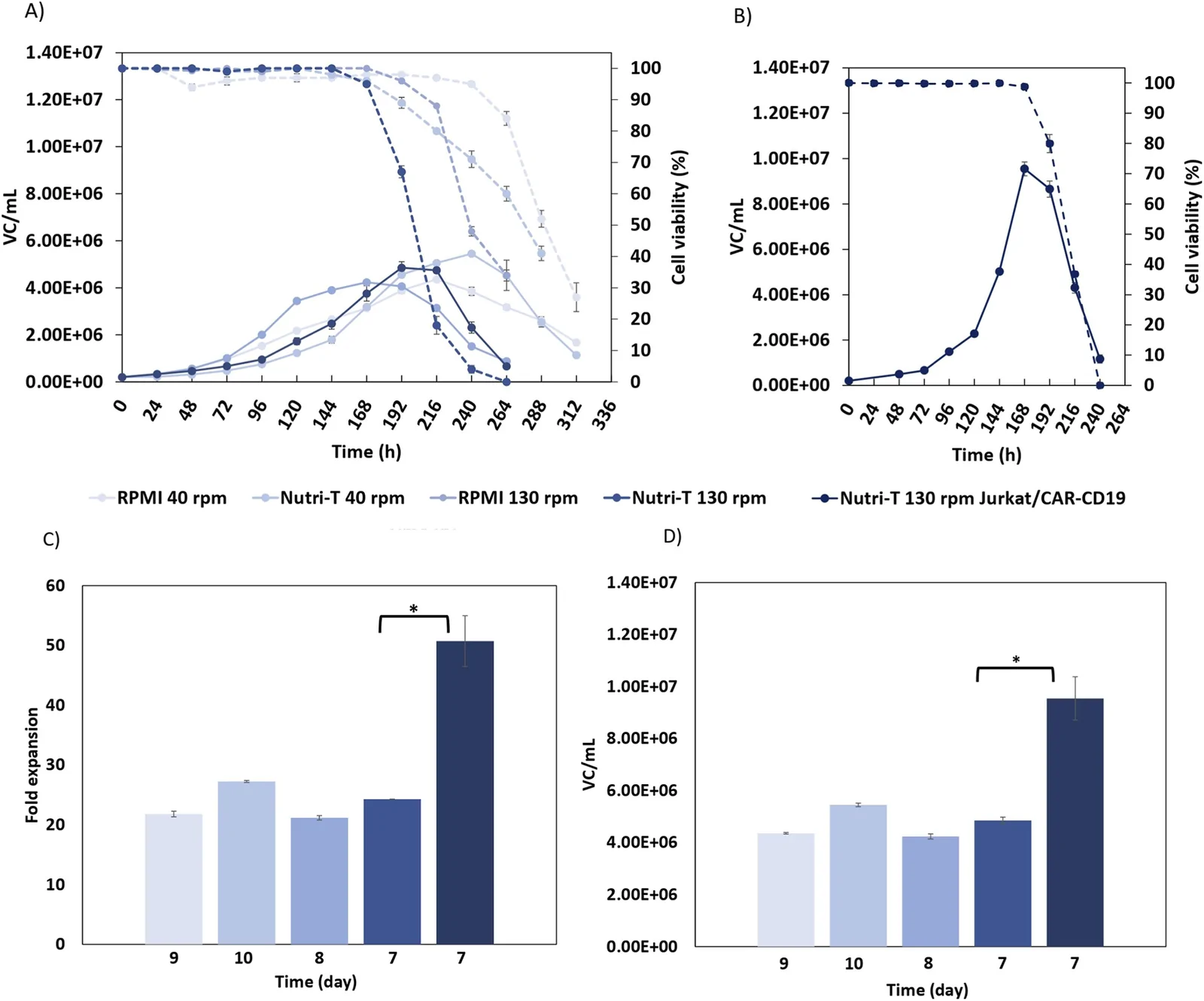
Effect of stirred batch growth kinetics of parental Jurkat and Jurkat/CAR-CD19 cell lines. **(A)** Growth kinetics in batch culture of parental Jurkat cells under two stirring conditions and two culture media. **(B)** Batch growth kinetics of Jurkat/CAR-CD19 cell line under selected condition (Nutri-T 130 rpm). **(C)** Fold expansion rates of each batch culture condition of parental Jurkat cell line and selected condition in Jurkat CAR/CD19. **(D)** Maximum cellular densities obtained in each batch culture condition of parental Jurkat cell line and in selected condition in Jurkat/CAR-CD19. (VC/mL); Viable cells per milliliter. Dotted lines in respective colors represent cell viability (%). Cell expansion was analyzed by two-way ANOVA with Tukey’s *post hoc* test. No significant differences were detected among parental lineage culture conditions. 130 rpm + Nutri-T media was selected for further evaluation in a genetically modified lineage, which exhibited significantly higher expansion than the parental lineage under the same condition (*p < 0.05, unpaired Student’s t-test). Data are shown as mean ± SD from two biological replicates performed in technical triplicate.

In the other hand, regarding the effect of shaking on batch cultures, the expansion results obtained under two shaking conditions (40, and 130 rpm) showed variations in growth phases [i.e., in differences in specific growth rates, μ, and doubling times, (td) and Dmax reached]. Under shaking conditions, the greatest cell expansion corresponded to Nutri-T medium at 40 and 130 rpm, reaching cell densities of 5.45 ± 0.069 × 10^6^ VC/mL and 4.86 ± 0.26 × 10^6^ VC/mL respectively (Figure 2D). These results correspond to their respective fold expansions values of 27.25 ± 0.16 and 24.28 ± 0.37 for 40 and 130 rpm in Nutri-T media respectively (Figure 2C). In relation to their specific growth rates (μ) lower values were observed compared to static at 40 and 130 rpm, with values of 0.0195 h^-1^ (td = 35.68 ± 3.11 h) and 0.0178 h^-1^ (td = 38.83 ± 0.76 h), respectively. This is reflected in a shift on the time required to reach Dmax, occurring at 240 h for 40 rpm and at 192 h for 130 rpm.

The results of the growth kinetics with the Jurkat/CAR-CD19 lineage showed that the Nutri-T medium further favors cell proliferation compared to what was previously observed in the parental lineage (Figure 2A) despite having been evaluated under the same conditions. Under the static 130 rpm shaking condition, a Dmax of 9.55 ± 0.36 × 10^6^ VC/mL (Figure 2B) was obtained along with a doubling time of 23.43 ± 0.21 h. The Dmax. was achieved after 168 h of culture, with cell viability remaining above 95% during this period. These results confirm the high expansion capacity of the Jurkat/CAR-CD19 lineage under batch culture condition with shaking at 130 rpm using Nutri-T medium. The comparison of the obtained kinetic parameters is shown in Supplementary Table S1.

### Metabolite analysis of batch growth kinetics

Figures 3A,C show the differences in initial glucose and glutamine concentrations between Nutri-T and RPMI media. Nutri-T medium showed a significantly higher basal glucose concentration, approximately 42.0 mmol/L, compared to 25.0 mmol/L for RPMI medium. Glucose uptake was also greater in Nutri-T than obtained in RPMI with a more pronounced decrease being observed between 72 and 144 h. This result correlates with the above-mentioned superior growth in this medium as well as their respective glucose consumption rate in Table 1, being these values statistically significant compared to the ones obtained in RPMI. At the end of the growth kinetics (240 h), the glucose concentration in Nutri-T medium was approximately halved, reaching 20.0 mmol/L, while in RPMI medium the reduction less pronounced, falling to 17 mmol/L. Initial glutamine uptake concentrations at the start of culture (0 h), the Nutri-T medium recorded a concentration of 4 mmol/L, compared to 1.13 mmol/L in the RPMI medium. In the Nutri-T medium, the glutamine concentration progressively decreased to 1.12 mmol/L, with its greatest reduction recorded between 72 and 144 h. In contrast, in the RPMI medium, glutamine was completely depleted from 144 h onward, with a marked decrease between 96 and 120 h. Contrary to these findings, differences in glucose or glutamine consumptions between cell lines (Jurkat parental and Jurkat/CAR-CD19) in the same media were not observed.

**FIGURE 3:**
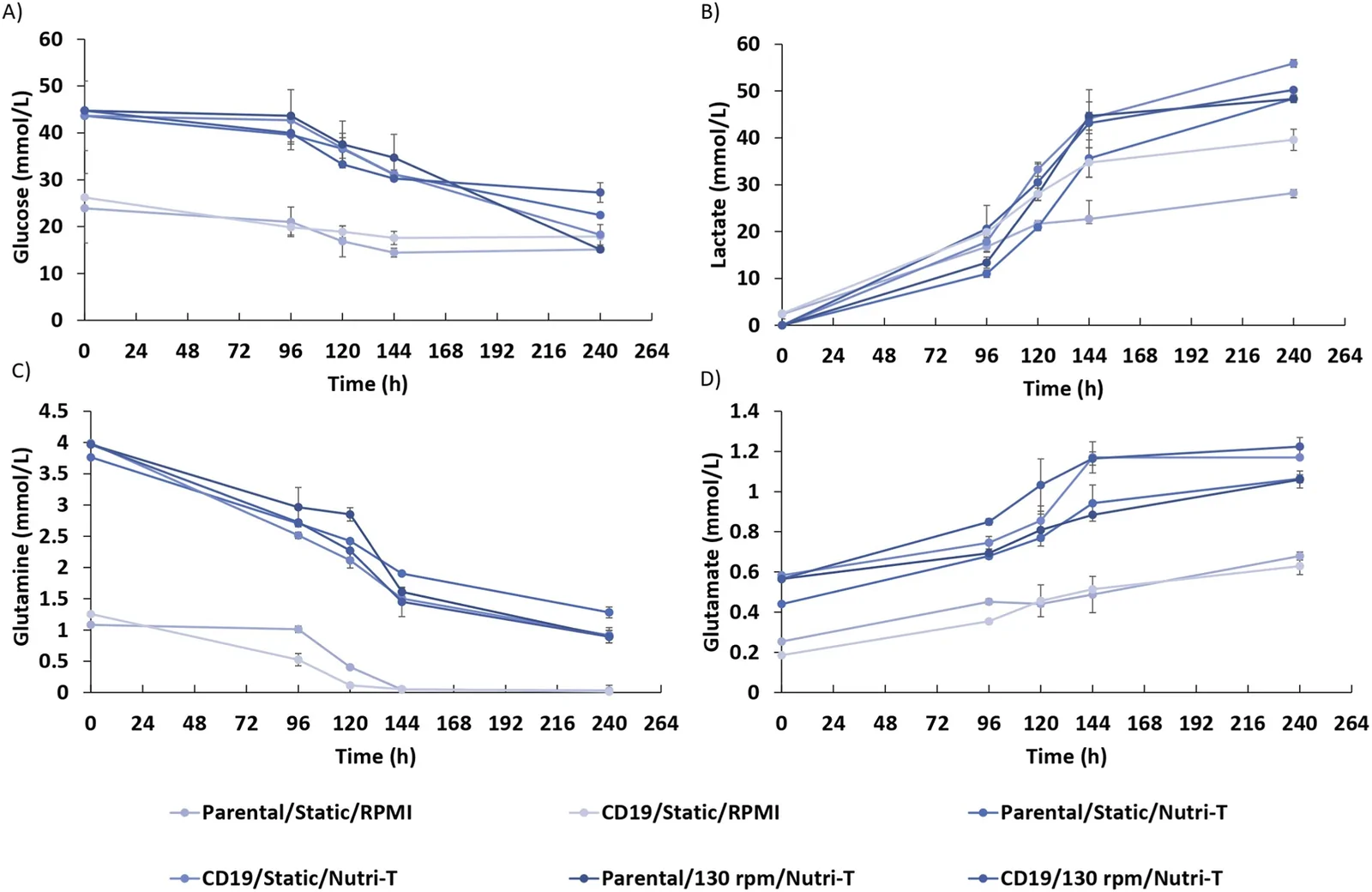
Comparative metabolite analysis of batch growth kinetics between Jurkat parental and Jurkat/CAR-CD19 cell lines. **(A)** Glucose uptake profile. **(B)** Lactate production profile. **(C)** Glutamine uptake profile. **(D)** Glutamate production profile.

**TABLE 1:** Specific rates of metabolite consumption and production in batch growth kinetics of parental Jurkat cell line.

Stirring state	Media	Cell line	qsGlu (pmol/cell*day)	qsGln (pmol/cell*day)	qpLac (pmol/cell*day)	qpE (pmol/cell*day)
Static	RPMI + 10%FBS	Jurkat	2.25 ± 0.08	0.18 ± 0.04	2.66 ± 0.03	0.05 ± 0.003
Static	RPMI + 10%FBS	CD19	1.96 ± 0.05	0.18 ± 0.03	3.6 ± 0.11	0.04 ± 0.006
Static	Nutri-T	Jurkat	4.05 ± 0.18 *	0.22 ± 0.03	6.52 ± 0.29 ***	0.13 ± 0.01
Static	Nutri-T	CD19	3.66 ± 0.04*	0.27 ± 0.01	4.66 ± 0.39 ***	0.14 ± 0.01
130 rpm	Nutri-T	Jurkat	2.37 ± 0.12	0.14 ± 0.01	1.91 ± 0.22	0.09 ± 0.005
130 rpm	Nutri-T	CD19	3.95 ± 0.34	0.27 ± 0.01	6.12 ± 0.05	0.13 ± 0.02

Statistical analysis of specific glucose consumption and lactate production rates was performed using two separate one-way ANOVAs followed by Tukey’s multiple comparisons tests. In the first analysis, RPMI + 10% FBS and Nutri-T were compared within each cell lineage under static conditions. In the second analysis, the effect of agitation was evaluated by comparing static culture and 130 rpm conditions in Nutri-T media within each lineage. *p<0.05 ***p<0.001.

Regarding the waste metabolite results, Figures 3B,D show the increase in lactate and glutamate concentrations respectively as growth kinetics progress. Figure 3B shows greater lactate accumulation in cultures grown in Nutri-T medium, reaching final values of 55 mmol/L for Jurkat/CAR-CD19 and 50 mmol/L for Jurkat. Harmonically, these results convey with the lactate specific production rate.

In RPMI medium, final lactate concentrations were lower, reaching 37 mmol/L for Jurkat/CAR-CD19 and 25 mmol/L for parental Jurkat. Figure 3D shows the accumulation of glutamate throughout the growth kinetics. With Nutri-T medium, an increase in glutamate concentration was obtained from 0.5 mmol/L to 1.1 mmol/L, whereas in growth kinetics with RPMI medium, the increase was smaller, going from 0.2 mmol/L to 0.5 mmol/L. Based on these results and supported by previous reports in the literature (as discussed later), glutamine was identified as the limiting metabolite, with concentrations of 0 mmol/L in RPMI and 1.5 mmol/L in Nutri-T at the critical time point of 144 h (late exponential phase). Additionally, lactate was determined to act as the excess metabolite, reaching a concentration between 30 and 40 mmol/L at 144 h in both culture media.

Table 1 presents the specific rates of metabolite consumption and production determined for static culture conditions and at 130 rpm, using both culture media (Nutri-T and RPMI) and both cell lines (Jurkat parental and Jurkat/CAR-CD19). The objective of this analysis was to support, from a metabolic perspective, the variations observed in the growth kinetics previously performed, as well as to evaluate whether the insertion of the CAR-CD19 gene by transduction generated any differentiable metabolic effect compared to the parental line. First, significant differences (p ≤ 0.05) were identified in the specific rates of glucose consumption (qsGlu) between the evaluated media. In the RPMI medium, values ranged between 1.9 and 2.01 pmol/cell*day, while in the Nutri-T medium, higher values were recorded, ranging from 2 to 3.56 pmol/cell*day. This increase in Nutri-T medium was consistent in both cell lines and under both agitations set ups (static or 130 rpm), demonstrating a greater glycolytic capacity associated with the increase in cell growth observed compared to RPMI media. This behavior was congruently reflected in the specific rates of lactate production (qpLac), where the obtained values (range 3.77–5.07 pmol/cell*day) were significantly higher (p ≤ 0.001) than those recorded in RPMI (range 5.57–7.75 pmol/cell*day). On the other hand, in general, no significant differences were found when comparing the consumption and production rates of the four metabolites evaluated when comparing both lineages under the same condition. That is, when comparing qsGlu, qpLac, qsGln and qpGlu between the parental Jurkat lineage and Jurkat/CAR-CD19 cultured in the same medium (Nutri-T or RPMI) and at the same shaking set up (static or 130 rpm). Finally, in relation to the specific rates of glutamine consumption (qsGln) and glutamate production (qpE), no statistically significant differences were found between the different conditions evaluated. However, the values obtained are consistent with the metabolic profiles previously observed in Figures 3C,D, reaffirming the expected behavior based on the progressive consumption and depletion of glutamine, as well as the moderate accumulation of glutamate during the exponential phase of growth.

### Fed batch and semi-perfusion growth kinetics

In fed-batch growth kinetics, distinct effects were observed derived from the start feeding time of nutrient supplementation (Figure 4A). During the first 96 h, all growth kinetics presented similar behavior in their lag phase; however, after the feeding in each strategy, variations were identified in the subsequent growth phases. In the FB1 strategy, at the beginning of feeding, the usual onset of the exponential phase was not observed at 96 h, as reported in batch cultures. In contrast, a second adaptation phase was identified after each feeding. The onset of the exponential phase was recorded from 144 h onwards, which extended to 192 h, followed by a 24 h lag phase, and then entering the death phase from 216 h onwards, with a 50% decrease in the VC/mL density. The Dmax achieved under this condition was 7.9 ± 0.35 × 10^6^ VC/mL, with a doubling time of 33.64 ± 0.79 h, as shown in Supplementary Table S2 (ST2). In the FB2 strategy, where feedings started in the late exponential phase, the expected behavior of the exponential phase was maintained after 96 h. However, from the third feeding (168 h), a “*plateau*” effect in growth occurred, followed by a new exponential phase that started at 192 h and lasted for 48 h, reaching a Dmax. of 9.1 ± 0.32 × 10^6^ with a doubling time of 28.24 ± 0.73 h (ST2). Subsequently, a similar abrupt death phase described above occurred under the Nutri-T/130 rpm in batch mode, with a 30% decrease in VC/mL over 24 h (Figure 4B). Finally, for the FB3 strategy, where feedings began in the mid-exponential phase, a similar behavior to FB2 was observed, with the difference that the plateau effect formed 24 h earlier. From this point on, a new exponential phase began, which concluded at 192 h with a Dmax of 11.8 ± 0.35 × 10^6^ VC/mL and a doubling time of 31.58 ± 0.58 h (ST2). This strategy proved to be the most efficient, as it achieved the highest recorded Dmax, even surpassing batch conditions by approximately 2 × 10^6^ VC/mL. Finally, it is noteworthy that, regardless of the observed behavior over the different strategies, cell viability remained above 95% until reaching Dmax in each case.

**FIGURE 4:**
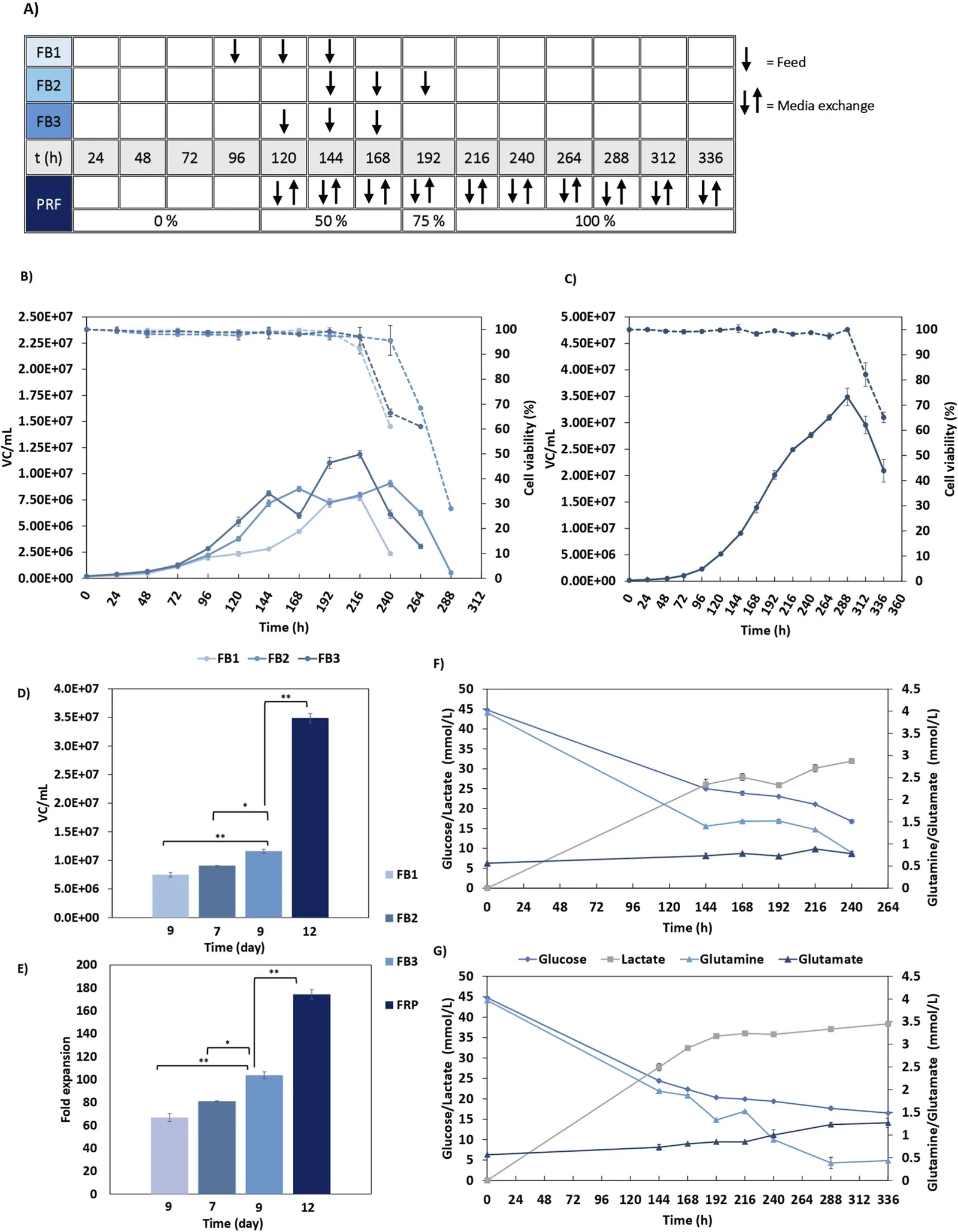
Fed-batch and semi-perfusion growth kinetics. **(A)** Addition and exchange strategies for fed batch and semi-perfusion growth kinetics **(B)** Fed-Batch growth kinetics under 130 rpm/Nutri-T condition **(C)** Semi-perfusion strategy under 130 rpm/Nutri-T condition **(D)** Maximum viable cellular densities obtained in fed-batch and semi-perfusion cultures in their respective days **(E)** Uptake and production metabolites profile in third fed-batch culture **(F)** Fold expansion rates of fed batch and semi-perfusion cultures **(G)** Uptake and production metabolites profile in semi-perfusion culture. Statistical analysis among fed-batch strategies was performed using one-way ANOVA followed by Tukey’s multiple comparisons test. Comparison between FB3 and semi-perfusion culture (PRF) was performed using an unpaired Student’s t-test. *p < 0.05, **p < 0.01. Data represents two independent biological replicates performed in technical triplicate.

To complement the comparative analysis between evaluated conditions together with viable cell concentration, total viable cell yield was calculated at the point of maximum viable cell concentration for each culture condition. The corresponding culture volume, total viable cell number, fold expansion, cumulative population doublings, and cumulative medium consumption are summarized in Supplementary Table S2.

Finally, in the semi-perfusion growth kinetics, a lag phase lasting 4 days was newly observed. Subsequently, at the beginning of the exponential phase at 120 h, the first media exchange was performed (Figure 4A), corresponding to 50% of the total volume, in accordance with the established criteria for reaching a cell density between 2 and 10 × 10^6^ VC/mL. This exchange set was maintained for 2 days until 168 h, at which point the exchange percentage increased to 75%, due to the cell density reaching 15 × 10^6^ VC/mL. According to the defined methodology (Figure 4A), once this value was exceeded, media exchanges were performed at 100%. Therefore, from 192 h to 336 h, daily changes of 100% of the media volume were performed. Throughout these exchanges, it was observed that, starting at 216 h, the specific growth rate decreased significantly, going from 0.031 h^-1^ (22.14 h) to 0.0047 h^-1^ (147.47 h) (ST2). This is reflected in the slope of the growth kinetics, which showed a lesser inclination during the exponential phase from that point onwards (Figure 4C). This decrease in the growth rate continued for the next 72 h, until reaching 288 h. From this point on, even with the daily 100% exchanges of media, a progressive decrease in cell viability was recorded, with an approximate loss of 20% per day. However, viability remained above 95% until 288 h, at which point Dmax was reached. This value corresponds to 3.5 ± 0.163 × 10^7^ VC/mL, almost three times higher than that obtained with the best fed-batch strategy (FB3 = 11.8 ± 0.35 × 10^6^ VC/mL). Furthermore, it is worth noting that, up to 192 h, the doubling time remained unchanged compared to the value described for the batch conditions in Nutri-T/130 rpm.

The metabolic profile for the fed-batch culture kinetics (Figure 4F) shows a progressive glucose consumption with a concentration decreasing from approximately 45 to 20 mmol/L as the exponential phase of growth progresses until 96 h. This same pattern was observed for glutamine, where its concentration decreases from 4 mmol/L to values between 1.5 and 2 mmol/L during the same period. Subsequently, the Figure 4F shows how glucose and glutamine concentrations stabilize from 96 h onwards due to the effect of the started daily feedings, remaining at relatively constant levels until 192 h. From this point on, a slight decrease in both concentrations is recorded, ending with values of <20 mmol/L for glucose and <1 mmol/L for glutamine, confirming the effectiveness of the feeding strategies in maintaining essential metabolite levels during the phase of greatest cell proliferation. Regarding waste metabolites, lactate production remained controlled throughout the kinetics, never exceeding 35 mmol/L in any of the three feeding strategies. It is important to highlight the behavior of the FB1 strategy, which did not exceed 20 mmol/L of lactate, an effect consistent with the delay in the onset of its exponential phase until 144 h, as shown in Figure 4B. Regarding glutamate, all three strategies maintained low production levels (<1 mmol/L) without significant increases throughout the culture kinetics. These results confirm the effectiveness of the feeding strategies implemented in controlling lactate accumulation, thus contributing to maintaining favorable conditions for cell expansion.

Regarding metabolic behavior in the semi-perfusion strategy (Figure 4G), a similar trend to that recorded in the three feeding strategies was initially observed. In the case of glucose, its concentration remained stable around 20 mmol/L throughout the culture phase, even until the cell death phase at 336 h. For glutamine, a progressive decrease was recorded, more pronounced than in the feeding strategies, reaching a final value of 0.5 mmol/L. Regarding waste metabolites, lactate production showed a behavior comparable to that observed in strategies FB2 and FB3 until 144 h. However, from that point on, its concentration continued to increase until 192 h, reaching values ​​higher than 35 mmol/L, which remained stable until 336 h. This behavior was expected considering the significantly higher cell density achieved in semi-perfusion mode (3.5 ± 0.16 10^7^ VC/mL) compared to the densities obtained in fed batches (7.9–11.8 × 10^6^ VC/mL). In the case of glutamate, its concentration remained comparable to the observed in fed batches until 216 h. Subsequently, during the following 72 h, a progressive increase was recorded, reaching values higher than 1.2 mmol/L. Overall, the obtained results indicate that the implemented semi-perfusion strategy was effective in maintaining adequate concentrations of consumer metabolites for an extended period, while largely limiting the increase in waste metabolites, even under the high cell densities achieved in the growth kinetics.

### Expression stability analysis

The CAR expression analysis is presented in Figure 5A shows the fluorescence profiles of the parental Jurkat cell line and the Jurkat/CAR-CD19 cell line used as fluorescence controls, together with the fluorescence distributions obtained from the experimental groups. Each experimental group represents the most efficient expansion condition identified for each mode of operation (fed-batch and semi-perfusion) and was evaluated at its respective late-exponential phase. The unstained Jurkat/CAR-CD19 population was used to define the baseline fluorescence distribution of the CAR-expressing cell line, whereas the parental Jurkat controls were used to characterize background fluorescence. Under the control conditions, fluorescence values were approximately 2 × 10^2^, whereas the experimental groups showed substantially higher fluorescence signals, reaching approximately 1 × 10^3^. The higher fluorescence intensity observed in the experimental groups indicates retention of detectable CAR-associated fluorescence at the evaluated late-exponential endpoint. In addition, two independent biological replicates were analyzed for each culture mode, and the fluorescence profiles showed similar patterns within each mode of operation.

**FIGURE 5:**
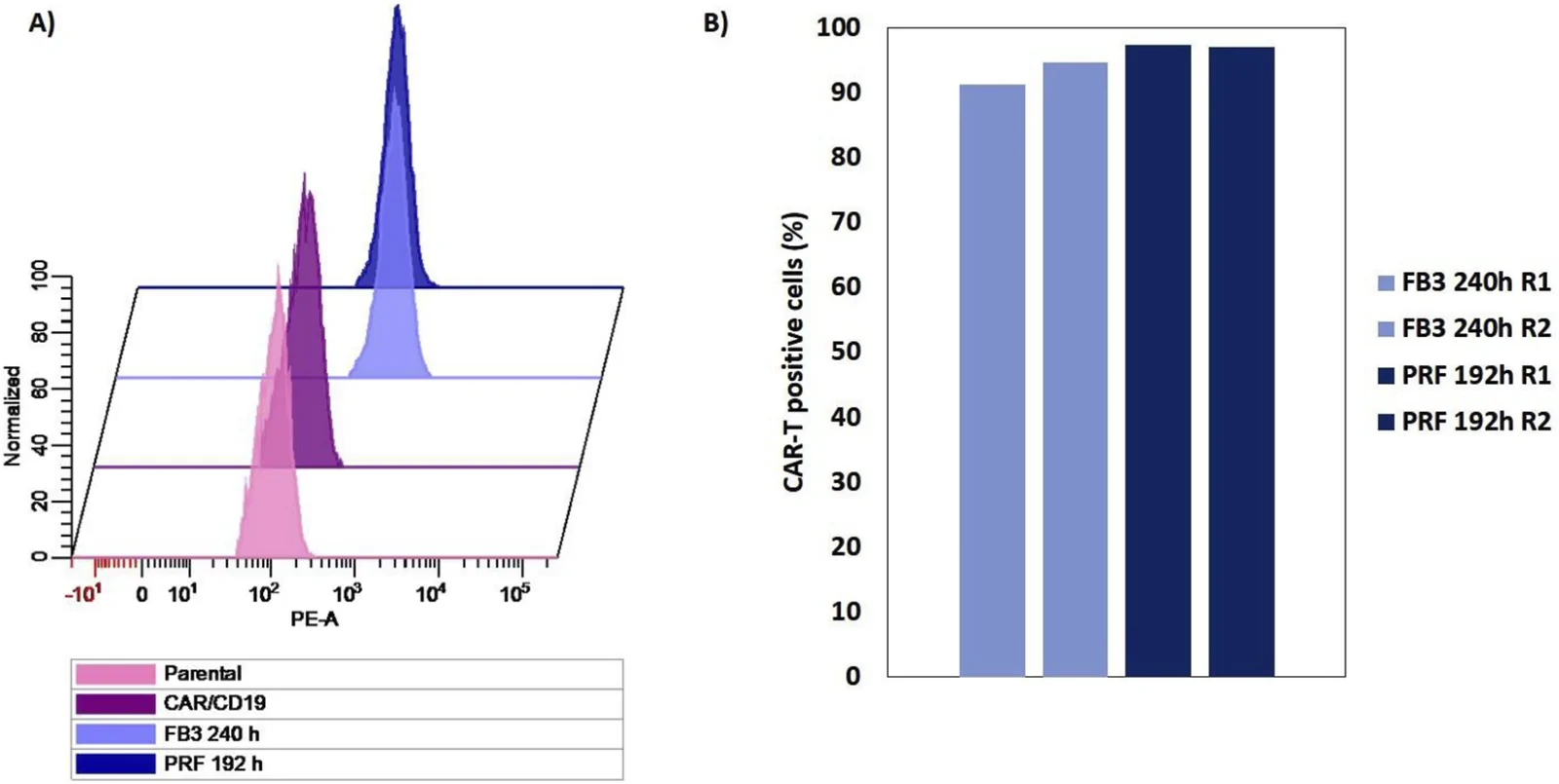
Expression stability of CAR-CD19 receptor under optimal culture conditions. **(A)** Overlay of expression levels of CAR-CD19 receptor on optimal expansion condition by mode of culture operation in their respective late-exponential phase. **(B)** CAR-T cell positive percentage by mode of culture operation in their respective late-exponential phase.

Figure 5B shows the corresponding CAR-positive populations, with both expansion strategies exhibiting high proportions of CAR-positive cells at the evaluated endpoint. The percentage of CAR-positive cells and the median fluorescence intensity (MFI) of the CAR-positive population were used as quantitative descriptors of CAR-associated fluorescence. The individual replicate values are presented in Supplementary Figure S2 to illustrate the consistency of the response within each culture strategy.

## Discussion

### Initial establishment of culture conditions for bioprocess optimization

Initial characterization of the Jurkat E6-1 model established the first set of culture conditions for subsequent bioprocess optimization. As outlined in the experimental workflow (Figure 1A), glucose availability (a fundamental component of the culture environment) was evaluated first. The markedly lower expansion observed with 2 g/L glucose, compared to 4.5 and 6.5 g/L, aligns with the strong reliance of proliferating T cells on glycolytic metabolism to meet their energy and biosynthetic needs. However, the lack of significant improvement in expansion when increasing the concentration from 4.5 to 6.5 g/L suggests that raising glucose availability beyond an intermediate level offered no additional proliferative advantage under the conditions tested. Furthermore, the highest glucose concentration was associated with slower growth kinetics and a more pronounced decline in viability, supporting the selection of 4.5 g/L as the reference glucose concentration for subsequent experiments. These observations are consistent with the principle that nutrient availability must be balanced against cellular metabolic demand, rather than simply maximized to increase substrate concentration (Zhu et al., 2021).

Following glucose-based selection, parental Jurkat E6-1 cells were adapted to Nutri-T medium (serum-free) to establish a more defined culture environment. The Nutri-T medium enabled a higher maximum viable cell density compared to RPMI medium under the conditions evaluated; however, this increase was associated with a longer doubling time and a marked decline in viability after peak density was reached. The greater expansion capacity observed with Nutri-T medium could be attributed to differences in the composition and availability of nutrients and other culture components compared to RPMI medium supplemented with FBS. Notably, the serum-free formulation also reduces reliance on serum supplements and provides a more defined culture environment for subsequent process development. These characteristics are relevant when evaluating culture conditions for advanced therapy manufacturing workflows, although this study does not establish equivalence between this model and the manufacturing of primary human T cells.

Subsequent comparison using Jurkat/CAR-CD19 cells demonstrated that the influence of culture conditions persisted after CAR introduction. With an initial inoculum of 1.0 × 10^5^ VC/mL, Nutri-T medium enabled a higher maximum viable cell density than RPMI, albeit with a longer doubling time and a more pronounced loss of viability. Increasing the initial inoculum to 2.0 × 10^5^ VC/mL further boosted the maximum viable cell density and improved apparent expansion kinetics. However, the rapid decline in viability after reaching peak density indicated that increasing the inoculum alone was insufficient to sustain culture performance during the final stages of expansion. These findings established a key foundation for the subsequent evaluation of agitation conditions and culture operations; aspects involving a more in-depth approach to controlling the cellular environment.

Overall, Figure 1 establishes a sequential and experimentally controlled strategy to identify culture conditions that can be translated to later stages of bioprocess development. Rather than defining an optimized manufacturing condition for CAR-T cells, these experiments establish a reproducible baseline framework using the CAR-CD19 Jurkat model, within which individual culture variables can be progressively evaluated and integrated. This approach aligns with the model’s intended use as an early-stage screening platform, while acknowledging that the resulting conditions require validation in primary human T cells before their applicability to CAR-T cell manufacturing can be determined.

### Comparative effect of type of medium cell cultures and stirring levels on batch mode on parental Jurkat lineage

To analyze the effect of medium cell culture type (RPMI and Nutri-T) and stirring levels (40 rpm and 130 rpm) an experimental matrix was made on parental Jurkat cell line. As presented in Figure 2A, these different combinations allowed the visualization of their respective effect on cell expansion. These results shed light on the characteristic growth behavior of the Jurkat cell line, identifying parameters such as its doubling time and the distinction between its different phases. In this way, it was possible to more accurately predict or estimate the duration of each of these phases. In the other hand, using the parental cell line allowed to establish optimal unaltered growth conditions, which served as a reference for later discerning the metabolic and/or proliferative effects derived from lentiviral transduction. This strategy also facilitated an initial screening of the most favorable culture conditions, which were subsequently applied to the Jurkat/CAR-CD19 lineage.

Regarding the results obtained from the growth kinetic combinations, Figure 2 showed that Nutri-T medium allowed higher cell concentrations to those obtained in RPMI medium. The highest cell density achieved in RPMI was 4.36 ± 0.28 × 10^6^ VC/mL, recorded under 40 rpm, while in the case of Nutri-T a higher value of 5.45 ± 0.69 × 10^6^ VC/mL was obtained. These statements correlate with previous work comparing Nutri-T and RPMI + 10% of human serum medium in primary CAR-T cells Anti/HER2 production, obtaining a superior cell expansion in Nutri-T (Yassin et al., 2025).

This higher growth observed in the Nutri-T culture medium could be explained by the higher basal levels of glutamine and other key proliferative components (IL-2, growth factors, etc.) in this medium compared with RPMI +10% FBS. This behavior on growth is observed in Figure 3A which shows greater availability and uptake of glutamine and glucose, these are essential metabolites specifically for lymphocytes cell growth given their elevated proliferation numbers (Tu et al., 2021). This hypothesis is reinforced by analyzing the specific glucose consumption rates reported in Figure 3, which shows that, in the Nutri-T/static condition with the parental lineage, the q_s_Glu = 4.05 ± 0.18 pmol/cell*day while in RPMI/static condition the qsGlu = 2.25 ± 0.08 being significative lower (p ≤ 0.05). This increased consumption rate on Nutri-T medium suggests greater glycolytic activity, which translates into a higher proliferative rate, reflected in the increases cell density reported in Figure 2. The towering role of the glycolytic pathway particularly in tumoral cell lines and activated lymphocytes in which the fast process is prioritized over the long-lasting but efficient process to obtain energy is commonly named as Warburg effect (Tu et al., 2021; Fernández-Ramos et al., 2017). In this phenomenon, it is expected to be superior glucose and glutamine uptake due to the demand of energy and biosynthetic precursors leaving the tricarboxylic acid process in second place. Moreover, a greater ATP production is also expected in a Jurkat model based on previous evidence while comparing it with primary T cells showing how Jurkat cell line purely relies in glycolytic pathway to proliferate (Ledderose et al., 2014). Together, with the mentioned results discussed in Figure 1 about the non-effect of higher levels of glucose, in this work it is suggested that superior concentrations of glucose beyond the mentioned threshold does not benefit the proliferation by itself but an intervention of and additional stimuli allow the exploitation of superior levels of this metabolite in the medium.

Another relevant aspect of the observed results in Figure 2A is the effect that different agitation levels had on cellular viability. Under agitation conditions of 40 and 130 rpm on Nutri-T medium, this parameter was observed to be prolonged. This extended viability is likely explained by the positive effect of agitation on nutrients and dissolved oxygen transfer, as well as by preventing the sedimentation and accumulation of waste products that can be toxic to cells. To validate this hypothesis, complementary studies are needed to evaluate such interactions as well as the resulting dissolved oxygen in each analyzed condition at different cellular concentrations.

### Observed growth differences on Jurkat/CAR-CD19 lineage on best batch conditions

Finally, based on the previously presented results, Nutri-T medium at 130 rpm was selected as the best option for testing in Jurkat/CAR-CD19 lineage due to his capability to reach the maximum cellular density earlier than the 40 rpm cell culture. Once executed, a cell density of 9.55 ± 0.36 × 10^6^ VC/mL was reached. Regarding doubling time, the obtained value was the fastest value on cell growth by batch. When comparing these cellular kinetic values with those obtained in the parental Jurkat lineage under the same culture conditions (5.98 and 4.86 × 10^6^ VC/mL respectively), a clear difference in the cell densities reached between the two lineages is evident. This difference suggests the possible presence of an effect induced by the lentiviral transduction used for the generation of the Jurkat/CAR-CD19 lineage, which would have enhanced its proliferative capacity specifically in the Nutri-T medium, as shown in Figure 2B. This phenomenon is known in the literature as the insertion effect, which refers to the phenotypic alterations caused by the aleatory integration of a gene into a cell’s genome, modifying its metabolism or proliferative behavior. This effect may be due to the inactivation, activation, or overexpression of genes close to the insertion site, considering that the *Retroviridae* family, to which lentiviruses belong, has a predilection for integrating its genetic material into active transcription regions within the genome (Mátrai et al., 2010). A possible scenario that would explain the greater growth observed in the Jurkat/CAR-CD19 lineage in Nutri-T medium, and not in serum-supplemented medium, is that the insertion of the CAR gene occurred in a promoter or regulatory region of genes encoding specific metabolic enzymes or transporters, whose substrate or limiting factor is only available in the Nutri-T formulation. This type of effect has been previously documented in cases where lentiviral integration has modified metabolic pathways dependent on certain medium components (Favor and Morawetz, 1992; Baum et al., 2006). Therefore, this insertion effect is considered as a possible determinant of the differential proliferation observed between the lines under the same culture conditions. Elucidating this phenomenon at the genomic and proteomic levels could provide further insight into the metabolic determinants underlying the differential proliferation of the Jurkat/CAR-CD19 model and help identify culture variables that may be relevant for future optimization studies in more physiologically representative CAR-T models.

### Development of fed Batch and semi-perfusion strategies based on metabolic measures from batch cultures

Since static culture conditions and 130 rpm in Nutri-T medium per batch demonstrated the greatest cell expansion capacity in both lineages, their metabolic profiles of metabolite consumption and production were analyzed (Figure 3). This analysis allowed us to identify potentially limiting nutriments and accumulated metabolic products, essential information for designing feeding strategies and medium changes in subsequent fed-batch and semi-perfusion experiments. An adequate identification of these critical metabolites was essential to avoid cell growth restrictions or toxic accumulations, thus improving the efficiency and stability of cultures under scalable conditions.

From this analysis (shown in Figure 3C), glutamine was identified as the limiting metabolite on Nutri-T media under the evaluated culture conditions, since its concentration decreased significantly during the late exponential phase (144 h), reaching values below < 2 mmol/L. This finding is relevant considering that glutamine plays an essential role in cell proliferation, not only as a nitrogen source for the synthesis of nucleotides, amino acids and other macromolecules, but also as a carbon source through its mitochondrial metabolism, directly contributing to the generation of ATP (Chen et al., 2025). Furthermore, in the case of activated T lymphocytes, such as the Jurkat lineage, this dependence is accentuated due to their high proliferation rates, which significantly increases the demand for glutamine-derived precursors. This effect was previously reported by Carr et al., in 2010, who demonstrated that glutamine concentrations below 2 mmol/L influence primary T lymphocytes proliferation, establishing a relationship directly dependent on the extracellular availability of this amino acid to sustain adequate cell expansion (Carr et al., 2010; Bopp et al., 2024).

On the other hand, lactate was identified as an excess metabolite, whose concentration increased markedly during the late exponential phase (144 h), exceeding 40 mmol/L. *In vitro* cultures can exert paradoxical effects depending on their concentration. Lactate contributes to redox balance by enabling NAD^+^ regeneration through lactate dehydrogenase (LDH), sustaining glycolysis and ATP production under hypoxia. Under normoxia, it can be reconverted to pyruvate and enter the Krebs cycle, but limited NAD^+^ availability in highly glycolytic cells restricts this pathway, favoring continuous lactate production even in oxygen-rich conditions (Kukurugya et al., 2025). Excess extracellular lactate acidifies the medium, impairing protein and enzyme activity. Notably, concentrations above 20 mmol/L compromise T cell activation, proliferation, and cytotoxicity (Fischer et al., 2007; Quinn et al., 2020; Rostamian et al., 2022).

### Effect of bolus supplementation in fed batch cultures

After identifying the limiting and excess media components in the batch kinetics, specific feeding strategies were designed (Figure 4A) with the aim of maintaining adequate glutamine concentrations, preventing it from becoming a limiting factor, and simultaneously diluting the accumulated lactate concentration in the culture medium.

This approach would allow the exponential phase to be prolonged, improving cell viability and achieving higher cell densities. To this end, three fed-batch strategies were established, differentiated by the timing of feeding initiation: during the early, mid, and late exponential phases. Although many studies consider the exponential phase as a continuous stage, in terms of cellular physiology, it is possible to distinguish critical moments within it, where nutritional needs and metabolic risks vary significantly. If these moments are not properly managed, metabolic limitations or waste accumulation can be generated that impair cell expansion.

The evaluated feeding strategies showed a common pattern characterized by the appearance of an adaptation phase after each supplementation bolus, attributed to the osmotic shock generated by the incorporation of fresh medium with a high concentration of nutrients. The FB3 strategy with the starting feeding at mid-exponential phase, showed better adaptation to the feeding effects and a greater expansion compared to the other two fed batch strategies. This behavior indicates that, when cells are in a more metabolically active state and with greater plasticity, their capacity to tolerate and overcome osmotic changes is greater, reducing the transient negative impact and favoring the continuation of proliferation. These findings are consistent with those described in CHO cultures, where bolus feedings have been associated with transient growth pauses before resumption of the exponential phase (Domján et al., 2020; Xiao et al., 2021), as well as with observations reported in CAR-T cell cultures, where transient growth pauses have also been described following the addition of relatively large volumes of fresh medium (Costariol et al., 2020). Together, these observations suggest that the timing of supplementation may influence the subsequent growth response, potentially due to differences in the metabolic state of the culture at the time of feeding (Mulukutla et al., 2015). In the present Jurkat/CAR-CD19 model, initiating supplementation at an earlier growth phase was associated with a more favorable expansion profile, although the underlying metabolic mechanisms were not directly evaluated.

### System bottleneck encountered during high-density semi-perfusion expansion

On the other hand, the applied semi-perfusion strategy showed growth almost three times greater than that obtained in the FB3, reaching a maximum density of 3.5 ± 0.16 × 10^7^ VC/mL with a sustained viability >95% at 288 h and a doubling time of 22.14 ± 0.30 h, positioning itself as the best expansion strategy developed in this work. As shown in Figure 4A, the strategy consisted of performing manual medium changes based on the density of viable cells obtained daily. As cell density increased, the percentage of medium change was progressively increased, keeping the cell pack constant and respecting the total working volume (25 mL). Figure 4G shows a slower decreased of glucose to the basal 45 mmol/L to 20 mmol/L at 288 h, while Nutri-T batch cultures on Figure 4C shows the same decreased at 240 h. This same concentration delay was observed on lactate, with the 40 mmol/L of lactate at 192 h while in batch cultures were observed at 144 h. The strategy aims to maintain constant concentrations of consumption and waste metabolites to favor both cell viability and sustained cell expansion.

Whereas, when higher the cell density is achieved, the greater the metabolic requirement and the production of byproducts, a larger replacement volume is required to avoid toxic accumulation and nutrient deficiencies. This principle explains why, once the threshold of 15 × 10^6^ VC/mL is exceeded as shown in Figure 4C, the recommendation for medium change may even be greater than 100% of the initial working volume in addition to the high cell density which could limit the adequate distribution of the dissolved oxygen. Regarding this last point, despite having made 100% changes in the medium volume starting from 192 to 288 h, a decrease in the doubling time was observed matching the starting daily lactate concentrations of 35 mmol/L (Figure 4G). This result suggests a diminished proliferation due to the produced change in the pH from the elevated lactate in the medium. This change in the pH medium could even affect the intermediaries on the glycolytic pathway, finally resulting in a deficient ATP production process. To maintain the energetic demand, it is possible that glutaminolysis was initiated at 240 h, in which time, an increase in glutamate concentration was observed (Figure 4G). Nevertheless, 48 h after the proliferation stopped. This metabolic deficient effect produced by the high cell density causes a bottleneck in the semi-perfusion system.

On the other hand, when considering the outcome of this semi-perfusion strategy from a manufacturing perspective, if the therapeutic dose required for clinical administration is reached at 144 h (2–5x10^8^ total cells), there would be no operational need to extend the culture to reach higher values ​​and possibly compromise the quality attributes of the final product. Furthermore, the flexibility in terms of production units of this expansion model allows the implementation of additional batches to obtain whether a backup dose to the same patient or to several more simultaneously (Watanabe et al., 2022). From a biotechnological process-development perspective, the expansion performance observed in this laboratory-scale Jurkat/CAR-CD19 model provides a basis for subsequent studies in more controlled culture systems. Future work could evaluate whether the identified expansion conditions can be reproduced in scalable platforms with controlled parameters such as dissolved oxygen, pH, and medium and metabolite exchange. Such studies would be particularly relevant for investigating the feasibility of higher-throughput expansion approaches, including their potential application to allogeneic CAR-T manufacturing. However, these possibilities remain prospective and were not evaluated in the present study.

### Expression stability of CAR-CD19 lineage in different modes of operation

CAR-CD19 receptor expression was evaluated at the late-exponential phase under the selected conditions for each operating mode. This phase was selected as the relevant endpoint for evaluating CAR expression because it represents a stage of active cell proliferation immediately preceding the transition toward the stationary phase, when changes in cell viability and growth kinetics may become apparent. Within the scope of the present laboratory-scale screening model, the results shown in Figure 5 indicate that the evaluated expansion strategies retain detectable CAR-CD19 expression at their respective late-exponential endpoints. Specifically, the high proportion of CAR-positive cells observed under the fed-batch and semi-perfusion conditions, together with the corresponding fluorescence intensity profiles, suggests that the evaluated expansion strategies did not result in an apparent loss of CAR expression at the assessed endpoint. However, this data should not be interpreted as evidence of longitudinal CAR-expression stability, since CAR expression was evaluated at a single time point for each selected condition. Longitudinal analyses from inoculation through harvest, together with functional assays, would be required to determine whether CAR expression and CAR-mediated activity are maintained throughout the culture process.

### A promising alternative for high-efficient CAR-T cell expansion

The increasing clinical implementation of CAR-T cell therapies has highlighted the importance of developing manufacturing processes that can support broader accessibility of these therapies. The high acquisition and treatment-related costs associated with currently approved CAR-T products remain an important challenge, particularly in healthcare settings where resources are limited (Wu et al., 2024; Cliff et al., 2023; Wu et al., 2024; Cliff et al., 2023). Within this context, optimization of the *ex vivo* expansion phase represents one of several process-development opportunities through which manufacturing performance may potentially be improved. However, reducing the cost of a clinical CAR-T product involves multiple factors beyond cell expansion alone, including materials, labor, equipment, quality control, logistics, and clinical administration.

In the present study, an early-stage laboratory screening model based on Jurkat/CAR-CD19 cells was used to compare different expansion strategies and identify culture conditions associated with improved proliferative performance. Using 125-mL shake flasks with a 25-mL working volume, agitation at 130 rpm, and serum-free Nutri-T medium, the selected culture condition achieved a maximum viable cell concentration of 3.5 ± 0.16 10^7^ VC/mL and a fold expansion of 174.4 ± 4.14 at day 12, while maintaining viability above 95%. These results demonstrate the capacity of the model to discriminate between culture strategies according to viable cell density, total viable cell yield, fold expansion, and culture duration.

To place these findings in the context of previously reported expansion approaches, the performance of the present model was compared with selected literature-reported strategies using different cell sources, culture media, and operating configurations (Supplementary Table S4). Such comparisons should be interpreted cautiously because the reported systems differ substantially in cell type, inoculation conditions, culture volume, feeding strategy, degree of process control, and culture platform. In particular, the present study was performed using an immortalized Jurkat/CAR-CD19 model in a laboratory-scale shake-flask system and therefore should not be considered directly equivalent to primary human CAR-T manufacturing processes or controlled bioreactor-based expansion platforms. Nevertheless, the observed expansion performance provides a useful benchmark for the early-stage evaluation of culture variables and process strategies in this model system.

Although some previously reported systems employ higher initial cell concentrations than the 2 × 10^5^ VC/mL used in the present study, fold expansion and culture duration provide complementary measures for comparing proliferative behavior across experimental systems (Costariol et al., 2020; Song et al., 2024; Hollyman et al., 2009; Hood et al., 2025; Sadeghi et al., 2011; Gatla et al., 2022; Zhang et al., 2018). In the present study, these metrics were used primarily to characterize the relative performance of the evaluated expansion strategies within the Jurkat/CAR-CD19 model rather than to establish direct manufacturing superiority over other platforms.

An additional advantage of the present approach is its experimental simplicity. The use of small-scale shake flasks and serum-free medium provides an accessible platform for systematic screening of culture variables while requiring substantially less specialized infrastructure than dedicated cell-manufacturing systems. However, this should not be interpreted as evidence of clinical cost-effectiveness. A rigorous economic assessment would require consideration of medium consumption, labor, equipment, quality control, consumables, contamination risk, process failure, and other manufacturing and clinical costs, which were beyond the scope of the present study. Likewise, although the use of a simple laboratory-scale format facilitates process exploration, scalability was not demonstrated and the present system should be regarded as a preliminary platform for subsequent scale-up investigation rather than as a scalable manufacturing process (O'Keeffe et al., 2025).

The main limitation of this study is the use of the immortalized Jurkat/CAR-CD19 cell line as a reductionist model. Jurkat cells provide high experimental reproducibility and tractability, allowing culture conditions and operating variables to be systematically screened while minimizing the biological variability associated with primary T-cell donors. However, they do not reproduce the full range of biological characteristics relevant to the actual primary CAR-T manufacturing, including donor-dependent responses, activation-dependent expansion, differentiation, memory phenotype, exhaustion, and other features of therapeutic T-cell products. Accordingly, the expansion strategies identified in this study should be interpreted as candidates for further evaluation rather than validated conditions for primary or for allogenic CAR-T manufacturing where the cell source will be more homogeneous and reproducible on its growth and metabolism allowing the generation of cellular products with consistent quality.

Within this framework, the repeated medium-exchange or semi-perfusion strategy achieved the highest viable cell density and total viable cell yield among the evaluated laboratory-scale conditions, identifying increased medium replacement as a promising process variable for further optimization. Nevertheless, this strategy required repeated centrifugation, cell resuspension, and manual medium replacement and therefore differs substantially from controlled perfusion systems that use continuous or semi-continuous medium flow and dedicated cell-retention mechanisms. These engineering differences, together with the absence of direct recovery and scale-up measurements in the present study, should be considered when interpreting the results and when designing future process-development studies. Nevertheless, it is important to consider that the bioreactor scale-up, does not represent a huge challenge and will decrease the cell manipulation and allowing a production out the hospitals that could be reflected in the cost of such products, mainly in non-developed countries.

Overall, the findings support the use of the Jurkat/CAR-CD19 system as an early-stage screening model for identifying expansion strategies and culture variables of interest before their evaluation in primary human CAR-T cells. The results provide a laboratory-scale benchmark and a basis for future studies aimed at determining whether the observed expansion performance can be reproduced in primary T-cell systems and subsequently translated to controlled and scalable manufacturing platforms.

## Conclusion

In this study, we established a reproducible CAR-CD19 Jurkat-based platform for systematic bioprocess development and optimization of cell expansion in a simplified laboratory-scale model. The sequential evaluation of glucose availability, culture medium, inoculum density, and operating conditions demonstrated that these variables substantially influence expansion kinetics, viable cell density, and culture viability. Furthermore, adaptation to the serum-free 4Cell Nutri-T medium enabled the assessment of a more defined culture environment while reducing reliance on serum supplementation. Notably, integrating growth and metabolic analyses provided additional insight into the relationship between culture conditions, nutrient utilization, and expansion efficiency.

Rather than serving as a substitute for primary CAR-T cells, the results demonstrate the utility of the Jurkat/CAR-CD19 system as an early-stage screening model for evaluating culture variables and expansion performance while minimizing biological variability and experimental complexity, enabling the systematic screening and integration of culture conditions.

Consequently, the resulting platform offers a laboratory-scale strategy for rational bioprocess development and for identifying conditions that can subsequently be evaluated and validated in primary CAR-T cells. Further studies using donor-derived T cells will be required to determine the extent to which the optimized conditions identified in this model are translatable to clinically relevant CAR-T manufacturing processes.

## Data Availability

The original contributions presented in the study are included in the article/Supplementary Material, further inquiries can be directed to the corresponding authors.
