# Supplementary material for: A CAR/CD19 Jurkat cell model for bioprocess screening and expansion: potential use for CAR-T cells production
Source: Front Bioeng Biotechnol. 2026 Oct 7;14:1917849. doi: 10.3389/fbioe.2026.1917849 (PMC13683766; doi:10.3389/fbioe.2026.1917849)
Supplement: Supplementary file 1 [file DataSheet1.docx]

**Supplementary** **Material**

**Supplementary Table 1.** Comparison of growth kinetic parameters obtained in Jurkat E6.1 and Jurkat/CAR-CD19 cell lines under batch mode by testing levels of agitation and two types of medium (RPMI + 10% FBS and Nutri-T).

| **Stirring set** | **Media** | **Maximum cell density (VC/mL)** | **μ**  **(h^-1^)** | **Doubling time**  **(h)** | **^Fold expansion^** |
| --- | --- | --- | --- | --- | --- |
| **Parental Jurkat** | | | | | |
| Static | RPMI + 10%FBS | 4.64 ± 0.10 x10^6^ | 0.0244 ± 0.002 | 28.47± 1.98 | 23.18 ± 0.11 |
| Static | Nutri-T | 5.98 ± 6.99 x10^6^ | 0.0299 ± 0.001 | 23.21 ± 1.20 | 29.92 ± 0.40 |
| 40 rpm | RPMI + 10%FBS | 4.36 ± 2.83 x10^6^ | 0.0222 ± 0.001 | 31.24 ± 1.19 | 21.8 ± 0.47 |
| 40 rpm | Nutri-T | 5.45 ± 6.90 x10^6^ | 0.0195 ± 0.001 | 35.68 ± 3.11 | 27.25 ± 0.16 |
| 130 rpm | RPMI + 10%FBS | 4.21 ± 9.81 x10^6^ | 0.0259 ± 0.0002 | 26.7 ± 0.29 | 21.18 ± 0.37 |
| 130 rpm | Nutri-T | 4.86± 2.58 x10^6^ | 0.0178 ± 0.0003 | 38.83 ± 0.76 | 24.28 ± 0.37 |
| **Jurkat CAR/CD19** | | | | | |
| Static | Nutri-T | 8.86x10^6^ ± 6.85x10^5^ | 0.0315 ± 0.0005 | 22.01 ± 0.39 | 44.3 ± 0.47 |
| 130 rpm | Nutri-T | 9.55x10^6^ ± 3.61x10^5^ | 0.0307 ± 0.0002 | 22.43 ± 0.21 | 50.73 ± 4.24 |

**Supplementary Table 2**. Comparison of growth kinetic parameters obtained in Jurkat/CAR-CD19 cell line under fed batch and perfusion cultures. B; Batch. FB; Fed Batch. PRF; semi -perfusion.

| **Operational mode** | **B1** | **FB1** | **FB2** | **FB3** | **PRF** |
| --- | --- | --- | --- | --- | --- |
| **Initial volume**  **(mL)** | 25 | 14 | 14 | 14 | 25 |
| **Initial Viable cells**  **(VC)** | 5.0×10⁶ | 2.8×10⁶ | 2.8×10⁶ | 2.8×10⁶ | 5.0×10⁶ |
| **Final volume**  **(mL)** | 25 | 25 | 25 | 25 | 25 |
| **Maximum cell density (VC/mL)** | 9.55 ± 0.36x10^6^ | 7.9 ± 0.35 x10^6^ | 9.1 ± 0.32 x10^6^ | 11.8 ± 0.35 10^6^ | 3.5 ± 0.16 10^7^ |
| **Maximum total viable cells**  **(TC)** | 2.38x10^8^ | 1.97x10^8^ | 2.27x10^8^ | 2.95x10^8^ | 8.75x10^8^ |
| **μ**  **(h^-1^)** | 0.0307 ± 0.0002 | 0.0198 ± 0.0002 | 0.0245 ± 0.001 | 0.0219 ± 0.0003 | 0.0313 ± 0.0004 |
| **Doubling time**  **(h)** | 22.43 ± 0.21 | 35.01 ± 0.50 | 28.24 ± 0.73 | 31.58 ± 0.58 | 22.14 ± 0.30 |
| **Fold Expansion** | 50.73 ± 4.24 | 66.84 ± 3.45 | 81.01 ± 0.42 | 103.7 ± 3.03 | 174.4 ± 4.14 |
| **Cumulative population doublings** | 5.66 ± 0.12 | 6.06 ± 0.07 | 6.34 ± 0.01 | 6.69 ± 0.04 | 7.44 ± 0.03 |
| **Total**  **consumed**  **medium (mL)** | 25 | 25 | 25 | 25 | 206.5 |

**Supplementary Figure 1.** Gating strategy for the identification of CAR-positive Jurkat/CAR-CD19 cells.

**Figure S1. Flow cytometry gating strategy for the identification of CAR-positive Jurkat/CAR-CD19 cells.** Representative flow cytometry plots showing the sequential gating strategy used for CAR expression analysis. (A) FSC-A versus FSC-H plot showing singlet discrimination. (B) FSC-A versus SSC-A plot showing selection of the cellular population. (C) PE-A versus SSC-A plot showing the fluorescence distribution used for CAR-positive cell identification. (D) Final CAR-positive population defined according to the PE fluorescence distribution relative to the corresponding unstained control. The upper row shows the Jurkat/CAR-CD19 at 0 h control used to assess autofluorescence, whereas the lower row shows a representative semi-perfusion (PRF) culture at 192 h (PRF 192 h R1). Approximately 10,000 events were acquired per sample, and the corresponding percentages and event numbers are indicated for each gate.

**Supplementary Figure 2.** Flow-cytometric fluorescence profiles of Jurkat/CAR-CD19 cells under different culture conditions.

**Figure S2. Flow-cytometric fluorescence profiles of Jurkat/CAR-CD19 cells under different culture conditions.** Representative one-dimensional PE fluorescence histograms showing the fluorescence distributions of parental Jurkat cells, unstained Jurkat/CAR-CD19 cells at 0 h, and Jurkat/CAR-CD19 cells cultured under fed-batch and semi-perfusion conditions. Fed-batch samples were analyzed at 240 h, whereas semi-perfusion samples were analyzed at 192 h. The same CAR-specific antibody and fluorescence channel were used for the analyzed samples. The unstained Jurkat/CAR-CD19 population was included as an autofluorescence control. The two biological replicates for each culture condition are shown individually to illustrate the reproducibility of the fluorescence profiles.

**Supplementary Table 3.** Quantitative flow-cytometric assessment of CAR expression in Jurkat/CAR-CD19 cells.

**Table S3. Quantitative flow-cytometric assessment of CAR expression in Jurkat/CAR-CD19 cells.** Flow-cytometric parameters obtained from the original FCS files for the biological replicates analyzed under fed-batch and semi-perfusion conditions. The table reports the total number of acquired events, events retained after singlet and cellular-population gating, number of CAR-positive events, percentage of CAR-positive cells, and median PE-A fluorescence intensity (MFI) of the CAR-positive population. Fed-batch samples were analyzed at 240 h and semi-perfusion samples at 192 h. Values are reported for each biological replicate individually.

| **Group** | **Acquired events** | **FSC/SSC-defined cellular population** | **CAR+ events** | **CAR %** | **MFI** |
| --- | --- | --- | --- | --- | --- |
| **Jurkat parental** | 10126 | 8556 | - | - | 64.61 |
| **Jurkat**  **CAR-CD19 0 h** | 10598 | 8676 | - | - | 51.87 |
| **FB3 240 h R1** | 12777 | 10477 | 9559 | 91.23 | 898.97 |
| **FB3 240 h R2** | 14278 | 10714 | 10148 | 94.71 | 1184 |
| **PRF 192 h R1** | 10884 | 9346 | 9102 | 97.38 | 528 |
| **PRF 192 h R2** | 10810 | 9193 | 8925 | 97.08 | 462.96 |

**Supplementary table 4.** Comparison of CAR-T cell expansion strategies in open, semi-open and closed systems.

| **Bioreactor** | **Initial seed (VC/mL)** | **Maximum cellular density (VC/mL)** | **Fold expansion** | **Cell Medium** | **Operation Mode** | **Cell linage** | **Reference** |
| --- | --- | --- | --- | --- | --- | --- | --- |
| **Culture Flask**  **(125 mL)** | 2x10^5^ | 35 x10^6^ | 174.4  (day 12) | Nutri-T  (Free serum) | Perfusion | Jurkat E6-1 | - |
| **WAVE**  **(bag 2 L, 6-15 rpm)** | 1.0x10^6^ | 13.2 –31 x10^6^ | 87 - 668  (day 13-18) | XVIVO-15 + 5% human serum (HS) + IL-2 | Perfusion (progressive volume exchange 200→1600 mL) | Primary  T-cells | [51] |
| **Xuri W-25**  **(bag 2 L)** | 5.0 x10^5^ | 1.07 x10^6^ | 265.4  (day 14) | TexMACS™  (Free serum) | Fed Batch | Primary  T-cells | [7] |
| **Ambr® 250**  **(300 rpm)** | 5.0 x10^5^ | 5.03 x10^6^ | ≈ 25.7  (day 7) | RPMI-1640 + 10% BFS | Perfusion (additions and exchanges ~50% medium since day 3) | Primary  T-cells | [4] |
| **Ambr® 250 (300 rpm)** | 5.0 x10^5^ | 20 x10^6^ | 85 (day 7) | RPMI-1640 + 10% BFS | Perfusion | Primary  T-cells | [52] |
| **WAVE** | 1.6 x10^5^ | 43 x10^6^ | 260 (day 14) | RPMI-1640 + 10% HS | Perfusion | Primary  T-cells | [53] |
| **Culture Flask**  **(static)** | 1.0E+06 | 25 x10^6^ | ≈ day 12.5 | XVIVO-15 + 5% human serum + IL-2 | Perfusion | Primary  T-cells | [54] |
| **CliniMACS**  **(250 mL)** | 4 x10^5^ | 6.4 x10^6^ | 16 (day 9) | TexMACS™  (Free serum) | Perfusion | Primary  T-cells | [55] |
| **CliniMACS**  **(250 mL)** | 2 x10^5^ | 19 x10^6^ | 94.7 (day 14) | Tex-CART  (Free serum) | Perfusion | Primary  T-cells | [7] |
